# Upper Eyelid Blepharoplasty: Surgical Techniques and Results—Systematic Review and Meta-analysis

**DOI:** 10.1007/s00266-023-03436-6

**Published:** 2023-07-10

**Authors:** Catarina Rodrigues, Francisco Carvalho, Marisa Marques

**Affiliations:** 1 Faculty of Medicine, University of Porto, Al. Prof. Hernâni Monteiro, 4200-319 Porto, Portugal; 2 Department of Plastic and Reconstructive Surgery, Centro Hospitalar de São João, Porto, Portugal; 3 Department of Surgery and Physiology, Faculty of Medicine, University of Porto, Porto, Portugal

**Keywords:** Upper blepharoplasty, Visual acuity, Dry eye, Intraocular pressure, Patient satisfaction, Complications

## Abstract

**Background:**

Upper eyelid blepharoplasty is a surgical procedure that aims to correct the typical changes that occur with aging to the periorbital area. The outcomes of this surgery are aesthetic, as well as functional. Many studies have described an impact on the cornea, intraocular pressure, dry eye syndrome, and visual quality. The aim of this systematic review is to compare the different surgical techniques and their outcomes.

**Methods:**

The authors performed a literature review through online databases PubMed, Web of Science, Clinicaltrials.gov, and CENTRAL libraries. Information was collected about the surgery techniques and the functional and aesthetic outcomes as well as complications of the interventions. Six types of upper blepharoplasty surgery were studied. Data were analyzed using Cochrane RevMan.

**Results:**

Twenty studies were included in our systematic review and nine in our meta-analysis. We presented results about intraocular pressure, central corneal thickness, flattest keratometry, steepest keratometry, corneal astigmatism, visual acuity, Schirmer test 1 and 2, tear film break-up time and the ocular surface disease index questionnaire, according to type of surgery. Our meta-analysis showed no significant results.

**Conclusions:**

No significant results were found; however, many studies reported an impact of upper blepharoplasty surgery in the outcomes studied. Only a small number of complications were reported, and patients were satisfied with the aesthetic outcomes.

**Level of Evidence III:**

This journal requires that authors assign a level of evidence to each article. For a full description of these Evidence-Based Medicine ratings, please refer to the Table of Contents or the online Instructions to Authors https://www.springer.com/00266.

## Introduction

The aging of the periorbita is characterized by a group of processes that can have an impact on eyelid aesthetics and function. Some typical changes that occur with aging are loss of volume in the upper eyelid and periorbita, a higher position of the eyelid crease, a lower position of the eyebrow, especially the lateral portion, reduction of skin elasticity [1], and dermatochalasis [2].

Dermatochalasis is a condition of the upper eyelid characterized by an excess of redundant skin [2]. Commonly associated with dermatochalasis is the selective loss and prolapse of the periorbital fat pads through the orbital septum [3, 4]. This condition has cosmetic as well as functional outcomes, such as the impact on visual acuity, inefficient eyelid elevation, and excessive use of the frontalis and orbicular muscles which can lead to discomfort and a stronger pronunciation of forehead rhytids [5, 6].

The upper eyelid has an anterior lamella, which includes the skin and the orbicularis muscle, and a posterior lamella, which includes the tarsal plate and the conjunctiva. Deep to the orbicularis muscle are located the nasal and central fat pads. Sometimes an accessory lateral fat pad can be found. These are different in texture and color. The nasal fat pad is located medially and is a lighter yellow in color while the central fat pad is located laterally to the nasal fat pad and is a darker yellow in color [4]. Deep to these fat pads is located the levator palpebrae superioris muscle that inserts into the tarsal plate and creates de superior eyelid crease [7].

Upper blepharoplasty surgery aims to treat dermatochalasis and achieve a more rejuvenated look to the periorbita by correcting the typical changes that occur with aging. The techniques of upper blepharoplasty surgery have evolved with time and several types of procedures can be done depending on the objective and the final look that is desired to be achieved [8, 9].

Firstly, blepharoplasty surgery can be done with resection of skin only [2, 6, 10, 11]. Another approach to this procedure is the resection of skin followed by the resection of a portion of the orbicularis oculi muscle as well [5, 12]. Another technique involves the resection of skin and herniated orbital fat, either from the central, the nasal, or from both fat pads. This can be done with or without resection of orbicularis muscle [8, 13–16, 18]. A distinct technique aiming for volume preservation in the upper eyelid can be performed by transposition and fixation of the nasal fat pad laterally, to the central eyelid area [9]. A different approach, with transposition of a pedicle of excess fat from the central fat pad to the lateral upper eyelid area, can be performed for addressing the loss of fullness in that area that occurs with aging [8]. Another technique that can be associated with blepharoplasty surgery is the brassiere suture where a suture is done from the lower and upper margins of the incision made on the orbicularis oculi muscle to the periosteum of the superolateral arcus marginalis. This suture is useful in repositioning the sub-brow fat pad and giving the eyebrow a lifted look [19].

Each surgery type has different outcomes that can be both positive and negative. While some outcomes are related to the aesthetic result and patient satisfaction with the final look, some studies have reported an impact on the cornea, visual acuity, intraocular pressure, and dry eye syndrome. With the development of such different approaches to this surgery, this systematic review aims to compare the different surgical techniques and their outcomes as well as discuss if certain results can be associated with upper blepharoplasty surgery or with a certain type of this procedure.

## Methods

This systematic review was conducted according to the Preferred Reporting Items for Systematic Reviews and Meta-Analyses (PRISMA).

### Study Selection

Two distinct database searches were performed for this study.

First, the authors searched for potentially relevant studies on PubMed, Web of Science, CENTRAL, and ClinicalTrials.gov electronic databases. The query “(blepharoplasty[MeSH Terms]) OR (eyelid/ surgery[MeSH Terms]) OR (blepharoplasty[Title/Abstract]) OR (dermatochalasis[MeSH Terms]) AND (upper[Title/Abstract])” was used for the PubMed database search. For the Web of Science search, the query “upper blepharoplasty (All Fields) or eyelid surgery (All Fields) and dermatochalasis (All Fields)” was used. The query “MeSH descriptor: [Blepharoplasty] explode all trees AND upper eyelid” was used for the CENTRAL search and, on ClinicalTrials.gov, the authors searched for the Condition or Disease: “Dermatochalasis” and Intervention/Treatment: “Blepharoplasty”.

The search was restricted to studies written in English and published in the *Plastic and Reconstructive Surgery*, *Aesthetic Surgery Journal*, and *Aesthetic Plastic Surgery* journals from January 2002 until October 2022. This restriction aimed to select good quality studies published in well-known journals with a high impact factor in plastic surgery, with a high amount of articles focused on aesthetic outcomes.

The eligibility assessment was carried out by two investigators independently. First, relevant studies were selected by title and abstract. Then, a more thorough selection was conducted by reading the full article. The selection was based on the inclusion and exclusion criteria primarily defined. Articles referred in the selected studies considered relevant to the study were also included.

Studies performed on patients who underwent upper blepharoplasty surgery for dermatochalasis or periorbital aging reasons were included. Exclusion criteria included patients with congenital malformations, ptosis, or Asian blepharoplasty.

The aim of this review was to study the positive and negative aesthetic outcomes of different types of upper eyelid blepharoplasty motivated by dermatochalasis or periorbital aging. The inclusion and exclusion criteria were designed to bring focus to this specific topic by excluding other possible reasons for upper eyelid blepharoplasty. The Asian population was excluded because of the surgical implications that the typical Asian eye shape and the Asian beauty standards would have, resulting in aesthetic outcomes that would not be comparable to those of the upper blepharoplasty techniques analyzed in this study.

The results obtained from this database search and selection were more qualitative in nature rather than quantitative, so, another database search was conducted.

For the second database search, the authors broadened the search criteria to include outcomes related to the impact of upper blepharoplasty surgery on intraocular pressure, dry eye syndrome, and corneal topography. Pubmed was searched using the queries “(blepharoplasty[MeSH Terms]) AND (intraocular pressure[MeSH Terms])”, “(blepharoplasty[MeSH Terms]) AND (corneal topograpy[MeSH Terms])” and “(blepharoplasty[MeSH Terms]) AND (dry eye syndrome[MeSH Terms])”. The queries used for Web of Science were “upper blepharoplasty (All Fields) and intraocular pressure (All Fields)”, “upper blepharoplasty (All Fields) and corneal topograpy (All Fields)” and “upper blepharoplasty (All Fields) and dry eye syndrome (All Fields)”. The queries used for the CENTRAL database search were “(MeSH descriptor: [Blepharoplasty] explode all trees) AND (MeSH descriptor: [Intraocular Pressure] explode all trees)”, “(MeSH descriptor: [Blepharoplasty] explode all trees) AND (MeSH descriptor: [Corneal Topography] explode all trees)”, and “(MeSH descriptor: [Blepharoplasty] explode all trees) AND (MeSH descriptor: [Dry Eye Syndromes] explode all trees)”.

The search was restricted to articles published in English from January 2002 until October 2022. Due to a lower number of results, the eligibility criteria for this second search did not restrict studies according to publication journals and the Asian population was included. Apart from this, the eligibility assessment was performed similarly to the first database search.

### Quality Assessment

Quality assessment was performed throughout the selection process. The risk of bias was calculated using the National Institute of Health. For most studies the *Quality Assessment Tool for Before-After (Pre-Post) Studies With No Control Group* was used where studies were classified as Poor if they scored 0-4, Fair if 5-8, and Good if 9-12. For two studies [11, 21] the *Quality Assessment Tool for Observational Cohort and Cross-Sectional Studies* was used and for another two studies [12, 22], the *Quality Assessment of Controlled Intervention Studies* was used. In both scales the article was considered Poor if it scored 0-4, Fine if 5-10, and Good if 11-14. For one study [23], the *Quality Assessment Tool for Case Series Studies* was used, where a study was considered Poor if it scored 0-3, Fine if 4-6, and Good if 7-9.

### Data Items

Data extraction was performed by one reviewer and then revised by the other two authors. Information about the type of upper blepharoplasty surgery performed, surgical outcomes, and complications was extracted.

The following types of blepharoplasty surgery were included: (1) upper blepharoplasty surgery with skin resection alone; (2) upper blepharoplasty surgery with skin and orbicularis oculi muscle resection; (3) upper blepharoplasty surgery with skin resection and transposition of a central fat pad pedicle; (4) upper blepharoplasty surgery with skin and nasal fat pad resection; (5) upper blepharoplasty surgery with skin, nasal and central fat pads resection; (6) upper blepharoplasty surgery with skin, orbicularis muscle, and nasal and central fat pads resection.

Data regarding the number of patients, number of eyes, reason for undergoing upper blepharoplasty surgery, severity of dermatochalasis, follow-up time, flattest keratometry (K1), steepest keratometry (K2), corneal astigmatism (CA), central corneal thickness (CCT), visual acuity, intraocular pressure (IOP), dry eye tests (Schirmer 1 and 2 tests, tear film break-up time [TF-BUT], ocular surface disease index questionnaire [OSDI]), aesthetic outcome, patient satisfaction, and complications were included.

Corneal topography and tomography are imaging techniques that analyze the shape and elevations of the cornea. Corneal topography describes the shape of the anterior surface of the cornea and corneal tomography performs a three-dimensional recreation of the anterior segment of the cornea while also providing information about the corneal thickness. These techniques describe the flattest (K1) and steepest (K2) meridians of the cornea which are used to calculate corneal astigmatism (CA). Corneal imaging techniques can also be used to measure central corneal thickness (CCT). CCT is a relevant factor to consider while assessing patients at risk for glaucoma since it can interfere with intraocular pressure (IOP) measurements [13, 24–28].

Intraocular pressure (IOP) can be calculated by tonometers. These estimate IOP by assessing the force needed to applanate the surface of the eye. The Goldmann applanation tonometry is considered the gold standard method for IOP measurement [29].

The Schirmer 1 and 2 tests and tear film break-up time (TF-BUT) are tests that can be performed to assess tear film production and stability. The Schirmer tests measure tear formation on a filter paper that is placed inside the lower eyelid. The difference between these tests is that the Schirmer 2 test is performed after the application of an anesthetic to the eye, measuring only the basal tear formation, while the Schirmer 1 test measures both the basal and reflex tear formation. Tear film break-up time (TF-BUT) is measured after the application of fluorescein into the tear film. The patients are asked to stop blinking and the TF-BUT is the time between a complete blink and the first break in the tear film [22, 30, 31].

The ocular surface disease index (OSDI) is a questionnaire formed of twelve questions used for patient assessment of dry eye symptoms [22].

### Synthesis Methods

Data were organized by outcome. A written description and comparison of results was performed. A meta-analysis including flattest (K1), and steepest (K2) keratometry, corneal astigmatism (CA), intraocular pressure (IOP), and tear film break-up time (TF-BUT) was performed. For the meta-analysis, the Cochrane RevMan Version 5.4.1 (Cochrane Collaboration, Copenhagen) was used. The data analyzed were continuous variables in the form of mean differences and standard deviations. For the meta-analysis, the results were analyzed in a random effects model. Heterogeneity was measured via the I^2^ test. A subgroup analysis was performed for skin only blepharoplasty and other surgery types. For all analyses *p* < 0,05 was considered significant.

## Results

**Fig. 1:**
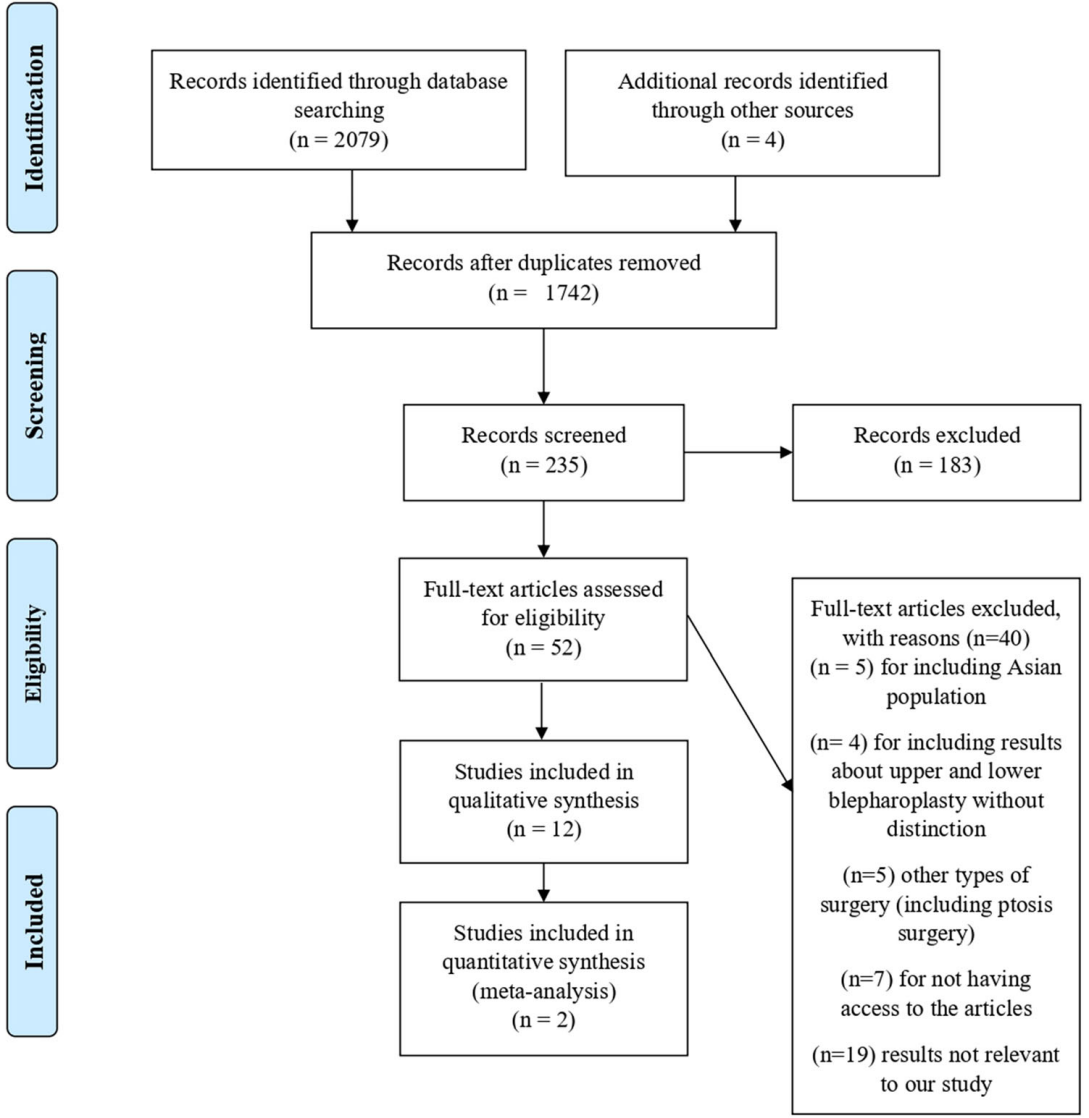
Flowchart representing the first database search according to PRISMA guidelines

Our first database search resulted in 2079 articles. After the selection process, 8 studies were selected for inclusion. Four relevant articles mentioned in the selected studies were also included. In total, 12 were selected for inclusion in our first database search. Figure 1 represents a flowchart of this selection process, according to PRISMA guidelines.

**Fig. 2:**
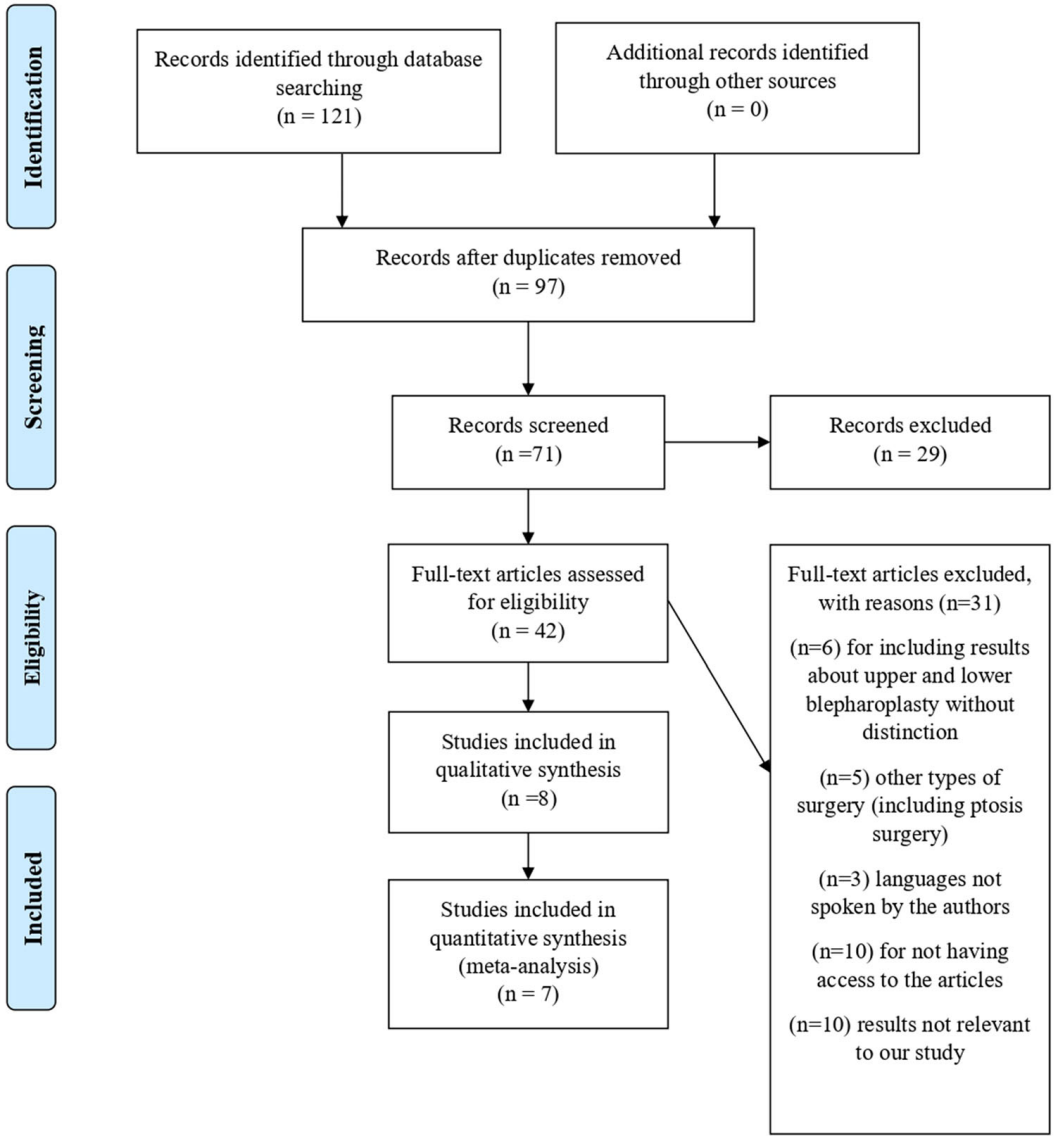
Flowchart representing the second database search according to PRISMA guidelines

Our second database search resulted in 121 articles. After the selection process, 8 studies were included. Figure 2 represents a flowchart of this selection process, according to PRISMA guidelines.

In total, 20 studies were included in our systematic review and 9 studies were included in our meta-analysis. All of them were considered to have fine or good quality. Within these studies, sixteen were prospective and four were retrospective. Nine studies presented results about skin only blepharoplasties, five about skin and orbicularis resection, one about skin and orbicularis resection with pedicled central fat pad, two about skin and nasal fat pad resection, two about skin, nasal, and central fat pad resection, one about skin, orbicularis, and nasal and central fat pad resection with orbicularis suture and three studies did not specify the type of blepharoplasty performed. Information about included studies can be found in Table 1. Information about the outcomes can be found in Table 2.

**Table 1:** Study characteristics

References	Risk of bias	Intervention	Follow-up time	Study type	Patients	Outcomes
Altin Ekin et al. [2]	Fair (7)	Skin only blepharoplasty	1 month	Prospective	103 patients (206 eyes)	CCT, K1, K2, CA, visual acuity, complications
Soares et al. [5]	Fair (7)	Skin and orbicularis resection	6 weeks	Prospective	14 patients (27 eyes)	Complications
Ilhan et al. [6]	Fair (7)	Skin only blepharoplasty	3 months	Prospective	56 patients (112 eyes)	IOP, CCT, K1, K2, CA
Sozer et al. [8]	Fair (5)	Skin and orbicularis resection with pedicled central fat pad	2 years	Retrospective	9 patients ^(a)^	Aesthetic outcome, complications
Nalci et al. [10]	Fair (7)	Skin only blepharoplasty	3 months	Prospective	34 patients ^(a)^	K1, K2, visual acuity,
Osaki et al. [11]	Fair (8)	Skin only blepharoplasty	6 weeks	Prospective	20 patients (40 eyes)	IOP, K1, CA, complications
Damasceno et al. [12]	Good (12)	Skin only blepharoplasty and skin and orbicularis resection	3 months	Prospective	15 patients ^(a)^	Aesthetic outcome, complications.
Zinkernagel et al. [13]	Fair (7)	Skin only blepharoplasty, skin and nasal fat pad resection, and skin, nasal, and central fat pad resection	3 months	Prospective	30 patients (58 eyes)	CCT, CA, complications
Bhattacharjee et al. [14]	Good (9)	Skin, nasal and central fat pad resection	1 year	Prospective	30 patients (60 eyes)	K1, K2
Ramella et al. [15]	Fair (5)	Skin and nasal fat pad resection	6 months	Prospective	30 patients ^(a)^	Aesthetic outcome, complications
Thomas et al. [16]	Fair (7)	Skin, orbicularis, and nasal and central fat pad resection with orbicularis suture	2 months	Retrospective	50 patients ^(a)^	Aesthetic result, complications
Aydemir et al. [17]	Fair (6)	Not specified	3 months	Prospective	20 patients (40 eyes)	TF-BUT
Vola et al. [20]	Fair (7)	Not specified	6 months	Prospective	30 patients (60 eyes)	K1, K2, CA
Mak, F. H. W. et al. [21]	Fair (8)	Skin and orbicularis resection	6-8 months	Prospective	7 patients (14 eyes)	TF-BUT, Schirmer 2 test, OSDI
Hollander et al. [22]	Good (14)	Skin and orbicularis resection	1 year	Prospective	54 patients (108 eyes)	TF-BUT, Schirmer 1 test, OSDI
Syniuta et al. [23]	Fair (6)	Skin only blepharoplasty	Case series	Retrospective	1 patient ^(a)^	Complications
Simsek et al. [32]	Fair (7)	Skin only blepharoplasty	3 months	Prospective	23 patients (43 eyes)	CA
Sommer et al. [33]	Fair (7)	Skin only blepharoplasty	1 month	Prospective	35 patients (42 eyes)	IOP, CA
Floegel et al. [34]	Fair (8)	Skin and orbicularis resection	3 months	Prospective	24 patients^(a)^	TF-BUT, Schirmer 1 test
Akidan et al. [36]	Fair (6)	Not specified	1 month	Retrospective	79 patients (158 eyes)	IOP

*(a)* Number of eyes not mentioned; *CA* Corneal astigmatism, *CCT* Central corneal thickness, *IOP* Intraocular pressure, *K1* Flattest keratometry, *K2* Steepest keratometry, *OSDI* Ocular surface disease index questionnaire, *TF-BUT* Tear film break-up time

**Table 2:** Study results

Outcome	Intervention	Significant difference	Non-significant difference
K1	Skin only	Increase: [6, 11]	Increase: [2, 10]
	Skin, nasal, and central fat pad resection		Decrease: [14]
	Not specified		Increase:[20]
K2	Skin only	Increase:[6] Decrease: [2]	Increase: [10]
	Skin, nasal, and central fat pad resection		Decrease: [14]
	Not specified	Increase: [20]	
CA	Skin only	Increase: [6, 11](for groups presenting with more severe dermatochalasis), [30] Decrease: [2]	Increase: [6] (for group presenting with milder dermatochalasis), [10, 13]^*^, [32]
	Skin and nasal fat pad		Increase: [13]^*^
	Skin, nasal, and central fat pad resection		Increase: [13]^*^
	Not specified	Increase: [20]	
Visual acuity	Skin only		Increase: [2] No difference: [10]
CCT	Skin only		Increase: [6] Decrease: [2] Not described:[13]^*^
	Skin and nasal fat pad resection		Not described: [13]^*^
	Skin, nasal, and central fat pad resection		Not described: [13]^*^
IOP	Skin only	Increase: [11]	Increase: [6, 32]
	Not specified		Decrease: [34]
Schirmer 1 test	Skin and orbicularis		Increase: [33] No difference: [22]
Schirmer 2 test	Skin and orbicularis		Increase: [21]
TF-BUT	Skin and orbicularis	Increase: [22] (6 months postoperatively)	Increase: [22] (12 months postoperatively), [33] Decrease: [21]
	Not specified		Increase:[17]
OSDI	Skin and orbicularis	Increase: [21] Decrease: [22]	

^*^Zinkernagel et al. [13] did not mention if the increase in CA was statistically significant in the groups individually so the results were considered non-significant

### Flattest Keratometry (K1)

Regarding skin only upper blepharoplasty, two studies found a significant increase in K1 values postoperatively [6, 11]. Two others found no statistically significant differences in this value [2, 10]. Ilhan et al. [6] found the variation of the keratometry values to be proportional to the severity of dermatochalasis presented preoperatively.

A study performing skin, nasal, and central fat pad resection found no significant difference in K1 [14].

### Steepest Keratometry (K2)

Among the studies that performed skin only upper blepharoplasty, one found a significant increase in K2, proportional to the severity of dermatochalasis presented preoperatively [6], one found a significant reduction of K2 [2] and another one found no significant differences [10].

No significant differences were found in patients who underwent skin, nasal, and central fat pad resection [14].

### Corneal Astigmatism (CA)

Regarding skin only resection, two studies showed a significant increase in CA [11, 30], and one study showed an increase of CA in forty-one percent of eyes [13]. One study showed a significant decrease in CA values [2]. Two articles showed no significant differences [10, 32]. Another article divided patients into groups by severity of dermatochalasis and showed a significant increase of CA in all, except for the group who presented with less severe dermatochalasis. The variation of this value was found to be proportional to the severity of dermatochalasis presented preoperatively [6].

An increase in CA values was found in fifty-seven percent of eyes in patients who underwent skin and nasal fat pad resection and in sixty-eight percent of patients who underwent skin, nasal, and central fat pad resection [13].

Zinkernagel et al. [13] found the increase in CA values to be significantly higher in patients who underwent upper blepharoplasty surgery with central and nasal fat pad resection when compared to patients who underwent skin only upper blepharoplasty.

### Central Corneal Thickness (CCT)

Three studies performing skin only upper blepharoplasty showed no statistically significant difference in CCT postoperatively [2, 6, 13].

One study performing both skin and nasal fat pad resection, and skin, nasal, and central fat resection, also found no significant difference in CCT scores after surgery [13].

### Visual Acuity

Two studies performing skin only blepharoplasty found no significant difference in visual acuity postoperatively [2, 10].

### Intraocular Pressure (IOP)

One study showed a significant increase in IOP after skin only upper blepharoplasty surgery [11], however, two other studies found no significant differences [6, 32].

### Tear Film Break-up Time (TF-BUT)

One study performing skin and orbicularis muscle resection found a significant increase in TF-BUT at six months postoperatively but not at twelve months [22]. Two other studies found no significant differences [21, 33].

### Schirmer 1 and 2 Tests

Two studies found no significant difference in the Schirmer 1 test [22, 33], and one study found no significant difference in the Schirmer 2 test [21] in patients who underwent skin and orbicularis muscle resection.

### Ocular Surface Disease Index Questionnaire (OSDI)

A significant increase in OSDI scores was found after upper blepharoplasty surgery with skin and orbicularis muscle resection by one study [21]. However, another article performing the same surgical technique found a significant decrease in the OSDI values postoperatively [22].

### Aesthetic Outcome

One study compared the aesthetic results of upper blepharoplasty with skin only resection and skin and orbicularis muscle resection. Three plastic surgeons scored the eyes on a scale from 0 (worst aesthetic result) to 10 (best aesthetic result) on the seventh, thirtieth, and ninetieth days postoperatively. Aesthetic scores were significantly higher in the skin only resection surgery group compared to those of the skin and orbicularis resection surgery group on the seventh day. No significant differences were found on the thirtieth and ninetieth days. All patients in this study were satisfied with the results [12].

In another article performing upper blepharoplasty with skin and orbicularis muscle resection patients also showed to be satisfied with the aesthetic results [21].

Sozer et al. [8] found that an upper blepharoplasty technique that performed skin and orbicularis resection with transposition of a pedicle of the central fat pad to the lateral upper eyelid area improved the lateral fullness of the periorbital area and achieved a more youthful look to the eyes. Both surgeons and patients showed satisfaction with the results and the increase in lateral volume remained stable during the two years follow-up time [8].

Ramella et al. [15] found all patients and surgeons to be satisfied with the postoperative symmetry of the eyes after skin and nasal fat resection upper blepharoplasty.

Thomas et al. [16] performed an upper blepharoplasty technique with skin, orbicularis, and nasal and central fat pad resection with orbicularis suture. Ninety-eight percent of patients were satisfied with this surgery. The other two percent represented a patient who wasn’t satisfied with the reduction of orbital fat. This issue was resolved after reintervention and the patient was satisfied with the results. This technique achieved a symmetrical definition of the crease in both eyes.

### Complications

One study compared skin only resection to skin and orbicularis muscle resection upper blepharoplasty. Patients rated edema, hematoma, itching, and pain from absent to severe on the seventh, thirtieth, and ninetieth days postoperatively. On the seventh day postoperatively, these results showed to be significantly lower in the skin only upper blepharoplasty surgery group. No significant differences were found on the thirtieth and ninetieth days. Other than these symptoms, there were no other postoperative complications [12].

Syniuta et al. [23] presented a case of a patient who developed strabismus with superior oblique palsy following skin only upper blepharoplasty surgery.

No complications were found for upper blepharoplasty surgery with skin only resection only in other studies [2, 11, 13].

One study performing skin and orbicularis muscle resection mentioned eyelid swelling that resolved six weeks postoperatively [5].

No complications were described in articles performing skin and orbicularis resection with transposition of a pedicle of the central fat pad [8], skin and nasal fat pad resection [13, 15], skin, nasal, and central fat pad resection [13], and skin, orbicularis, and nasal and central fat pad resection with orbicularis suture [16].

### Meta-analysis

A meta-analysis was performed for the following outcomes: flattest keratometry (K1), steepest keratometry (K2), corneal astigmatism (CA), intraocular pressure (IOP), and tear film break-up time (TF-BUT) scores. The mean difference between these values preoperatively and postoperatively was evaluated.

Four studies [2, 11, 14, 20] were included in the K1 meta-analysis. No significant difference was found in K1 values when analyzing all studies (mean difference: 0.09 [  −  0.16; 0.33]; *P*=0.49). A specific analysis was performed for skin only blepharoplasties and no significant differences were found (two studies [2, 11], mean difference: 0.28 [− 0.16; 0.73]; *P*=0.21). No significant differences were found when an analysis was performed for a follow-up time of more than a month (three studies [11, 14, 20], mean difference: 0.01 [− 0.27; 0.29]; *P*=0.95) or for a follow-up time of six months or higher (two studies [14, 20], mean difference: 0.00 [− 0.30; 0.30]; *P*=0.99).

A subgroup analysis was performed comparing skin only upper blepharoplasties (two studies [2, 11]) with the other blepharoplasty techniques (two studies [14, 20]), and no significant differences in K1 were found between the subgroups (P=0,30). More information can be found in Table 3.

**Table 3:** Meta-analysis results for K1

Study	Number of eyes	Preoperative		Postoperative		
		Mean (D)	SD	Mean (D)	SD	Mean Difference
Altin Ekin et al. [2]	103	43.35	1.92	43.68	1.77	0.33 [− 0.17; 0.83]
Osaki et al. [11]	40	44.66	2.06	44.78	2.28	0.12 [− 0.83; 1.07]
Bhattacharjee et al. [14]	60	42.41	1.08	42.40	1.08	− 0.01 [− 0.40; 0.38]
Vola et al. [20]	60	43.95	1.30	43.96	1.29	0.01 [− 0.45; 0.47]
Total (95% CI): *P*=0.49; I^2^ = 0%						0.09 [− 0.16; 0.33]
Skin only blepharoplasties [2, 11] (95% CI): *P*=0.21; I^2^ = 0%						0.28 [− 0.16; 0.73]
Follow-up > 1 month [11, 14, 20](95% CI): *P*=0.95; I^2^ = 0%						0.01 [− 0.27; 0.29]
Follow-up ≥ 6 months [14, 20] (95% CI): *P*=0.99; I^2^ = 0%						0.00 [− 0.30; 0.30]
Subgroup differences (skin only blepharoplasty [2, 11] and other techniques [14, 20]): *P* = 0,30; I^2^ = 8,6%						

D: Diopters; SD: Standard deviation; 95% CI: Ninety-five percent confidence interval; I^2^: I^2^ test for heterogeneity; *P*: *P* values

Three studies were included in the K2 meta-analysis [2, 14, 20] and no significant differences were found between them (mean difference: − 0.20 [− 0.43; 0.04]; *P*=0.10).

A subgroup analysis was performed to compare skin only upper blepharoplasties (one study [2]) with the other blepharoplasty techniques (two studies [14, 20]) and no significant differences in K2 were found between subgroups (*P*=0.40). More information can be found in Table 4.

**Table 4:** Meta-analysis results for K2.

Study	Number of eyes	Preoperative		Postoperative		
		Mean (D)	SD	Mean (D)	SD	Mean Difference
Altin Ekin et al. [2]	206	44.45	1.83	44.14	1.79	− 0.31 [− 0.66; 0.04]
Bhattacharjee et al. [14]	60	42.30	1.05	42.06	1.05	− 0.24 [− 0.62; 0.14]
Vola et al. [20]	60	45.17	1.46	45.31	1.47	0.14 [− 0.38; 0.66]
Total (95% CI): *P*=0.10; I^2^ = 2%						− 0.20 [− 0.43; 0.04]
Subgroup differences (skin only blepharoplasty [2] and other techniques [14, 20]): *P* = 0.40; I^2^ = 0%						

D: Diopters; SD: Standard deviation; 95% CI: Ninety-five percent confidence interval; I^2^: I^2^ test for heterogeneity; *P*: *P* values

For the CA meta-analysis, five studies were included [2, 11, 20, 30, 32]. No significant differences were found when analyzing all studies (Mean difference: 0.01 [− 0.15; 0.17]; *P*=0.91). A specific analysis was performed for skin only upper blepharoplasties and no significant differences were found (four studies [2, 11, 30, 32], mean difference: 0.00 [− 0.19; 0.18]; *P*=0.97). Then, only studies that had a follow-up time of more than a month were analyzed and no significant differences were found (three studies [11, 20, 30], mean difference: 0.11 [− 0.04; 0.26]; *P*=0.16). No significant differences were found for a follow-up time of three months or higher as well (two studies [20, 30], mean difference: 0.11 [− 0.14; 0.36]; *P*=0.40). More information can be found in Table 5.

**Table 5:** Meta-analysis results for CA

Study	Number of eyes	Preoperative		Postoperative		
		Mean (D)	SD	Mean (D)	SD	Mean difference
Altin Ekin et al. [2]	206	1.01	1.30	0.79	0.71	− 0.22 [− 0.42; − 0.02]
Osaki et al. [11]	40	0.78	0.43	0.89	0,45	0.11 [− 0.08; 0.30]
Simsek et al. [32]	43	1.10	0.80	1.20	0,70	0.10 [− 0.22; 0.42]
Sommer et al. [33]	42	1.00	0.88	1.06	0,88	0.06 [− 0.32; 0.44]
Vola et al. [20]	60	1.22	1.14	1.34	1,16	0.12 [− 0.29; 0.53]
Total (95% CI): *P*=0.91; I^2^ = 39%						0.01 [− 0.15; 0.17]
Skin only blepharoplasties [2, 11, 30, 32] (95% CI): *P*=0.97; I^2^ = 52%						-0,00 [− 0.19; 0.18]
Follow-up > 1 month [11, 20, 30] (95% CI): *P*=0.16; I^2^ = 0%						0,11 [− 0.04; 0.26]
Follow-up ≥ 3 months [20, 30] (95% CI): *P*=0.40; I^2^ = 0%						0.11 [− 0.14; 0.36]

D: Diopters; SD: Standard deviation; 95% CI: Ninety-five percent confidence interval; I^2^: I^2^ test for heterogeneity; *P*: *P* values

Three studies were included in the IOP meta-analysis [11, 32, 34]. When all studies were analyzed, no statistically significant difference was found (mean difference 0.32 [− 0.37; 1.01]; *P*=0.36). When a specific analysis was performed for follow-up time, a non-significant increase in IOP values was found for a follow-up time of six weeks or higher (two studies included [11, 32], mean difference: 0.81 [− 0.09; 1.71]; *P*=0.08). More information can be found in Table 6.

**Table 6:** Meta-analysis results for IOP.

Study	Number of eyes	Preoperative		Postoperative		
		Mean (mmHg)	SD	Mean (mmHg)	SD	Mean Difference
Akidan et al. [36]	158	15.562	2.629	15.543	2.673	− 0.02 [− 0.60; 0.57]
Osaki et al. [11]	40	14.190	2.120	15.210	2.600	1,02 [− 0.02; 2.06]
Sommer et al. [33]	42	16.300	4.100	16.500	4.300	0.20 [− 1.60; 2.00]
Total (95% CI): *P*=0.36; I^2^ =31%						0.32 [− 0.37; 1.01]
Follow-up ≥ 6 weeks [11, 32] (95% CI): *P*=0.08; I^2^ = 0%						0,81 [− 0.09; 1.71]

SD: Standard deviation; 95% CI: Ninety-five percent confidence interval; I^2^: I^2^ test for heterogeneity; *P*: *P* values

Regarding TF-BUT scores, two studies were included in the meta-analysis [17, 33] and no significant differences were found (mean difference 0.55 [− 0.53; 1.62]; *P*=0.32). More information can be found in Table 7.

**Table 7:** Meta-analysis results for TF-BUT

Study	Number of patients	Preoperative		Postoperative		
		Mean (s)	SD	Mean (s)	SD	Mean difference
Aydemir et al. [17]	20	10.47	2.19	11.32	2.93	0.85 [− 0.75; 2.45]
Floegel et al. [34]	24	6.90	2.62	7.20	2.50	0,30 [− 1.15; 1.75]
Total (95% CI): *P*=0.32; I^2^ = 0%						0,55 [− 0.53; 1.62]

S: Seconds; SD: Standard deviation; 95% CI: Ninety-five percent confidence interval; I^2^: I^2^ test for heterogeneity; *P*: *P* values.

## Discussion

Many studies have reported that upper blepharoplasty surgery can have an impact on visual quality [2, 10, 14, 34]. Some articles suggest that the alterations in the pressure the eyelid exerts on the cornea after upper blepharoplasty surgery could cause changes in its shape and refractive characteristics [13, 20, 30, 35].

Corneal topography and tomography are imaging techniques that analyze the shape of the anterior cornea and its elevations measuring variables such as flattest keratometry (K1), steepest keratometry (K2), and corneal astigmatism (CA) [13, 24–27].

In our systematic review, six studies [2, 6, 10, 11, 14, 20] presented results regarding K1 values, five studies [2, 6, 10, 14, 20] regarding K2 values, and eight [2, 6, 10, 11, 13, 20, 30, 32] regarding CA values. The study characteristics can be found in Table 2.

Two studies [6, 11] reported a significant increase in K1 values postoperatively. However, our meta-analysis found no significant differences in K1 values when including all studies, skin only resection blepharoplasties, or different follow-up times. No significant differences were found between the skin only blepharoplasty group and the other surgical techniques.

Two studies [2,6, 20] found a significant increase in K2, and one study [2] reported a significant decrease in this value postoperatively. No significant differences were found for K2 values on our meta-analysis when including all studies, skin only resection blepharoplasties, and no significant differences were found between the skin only blepharoplasty group and the other types of surgery.

Four studies [ 6, 11, 20, 32] found a significant increase in CA. However, one [6] of these studies grouped patients by severity of dermatochalasis and found this increase to be significant in all groups except the one presenting with less severe dermatochalasis. One other study found a significant decrease in CA [2]. Our meta-analysis found no significant differences when including all studies, skin only resection surgery, and different follow-up times. No differences were found when comparing skin only blepharoplasties with the other techniques.

In this systematic review, two studies [2, 10] found no significant changes in visual acuity postoperatively. These results are concordant with the fact that no significant differences were found for K1, K2, and CA in our meta-analysis since important changes in these values could have an impact on visual acuity.

Ilhan et al. [6] found the increase of K1, K2, and CA values to be proportional to the severity of dermatochalasis presented preoperatively. Perhaps the reason for the lack of significant results in our analysis was not dividing patients into groups according to the severity of dermatochalasis. Such analysis could not be performed as most of the studies selected grouped all patients together.

However, Zinkernagel et al. [13] found changes in CA values to be more significant in surgeries with a reduction of the nasal and central fat pads. Therefore, the lack of significance in our meta-analysis results could also be justified by most of the studies included having performed skin only blepharoplasties.

Regarding the biomechanical proprieties of the eye, some studies have presented results about the impact of upper blepharoplasty surgery on IOP [6, 11, 32, 34] and CCT [2, 6, 13].

Intraocular pressure (IOP) is an important risk factor for open-angle glaucoma. The Goldmann applanation tonometry is the gold standard measurement for IOP. However, this measurement can be affected by central corneal thickness (CCT). A low CCT can lead to a falsely low IOP result and the contrary can happen with a high CCT. Moreover, some studies have suggested that CCT can be associated with a higher risk of developing glaucoma, even though the role CCT has on its progression and severity in patients with established glaucoma is still uncertain. [36–38].

Osaki et al. [11] suggested that the removal of excess skin causes the remaining eyelid skin to increase tension around the eye globe, which could be the cause of increased IOP. Ilhan et al. [6] demonstrated a non-significant increase in CCT values, higher in patients presenting with a more severe dermatochalasis. They stated that even though this increase was non-significant, patients presenting with a more severe dermatochalasis should have a closer follow-up postoperatively.

In our systematic review, three studies [6, 11, 32] investigated the impact this surgery has on IOP, being that one [11] of them reported a significant increase in IOP after skin only upper blepharoplasty surgery. Three studies [2, 6, 13] investigated the impact upper blepharoplasty surgery has on CCT and none of them presented significant results.

Our meta-analysis found no significant results for IOP when including all studies and when analyzing a follow-up of more than 6 weeks. No meta-analysis was performed for CCT values.

Our results suggest that upper blepharoplasty surgery doesn’t significantly impact IOP or CCT values. Nevertheless, only three studies were included in our IOP meta-analysis, and the number of studies included in our systematic review was also small. Besides that, taking into consideration the results presented by Ilhan et al. [6], perhaps grouping patients by severity of dermatochalasis could lead to significant results in groups presenting with a more severe dermatochalasis preoperatively. Analysis of results according to the type of surgery would also be an interesting approach. This analysis could not be performed due to lack of data.

Transient dry eye symptoms are commonly reported after upper blepharoplasty. These can sometimes be attributed to corneal irritation from the surgery and appear in the first week postoperatively [39, 40]. Other reasons for these symptoms are the incomplete palpebral closure that occurs in the immediate postoperative period and the exposition of the cornea that was previously covered by the excess skin to external factors such as wind. During this time, it is commonly recommended the use of eye lubricants and sunglasses, and taping the eyes to remain closed during sleep [41].

The orbicularis oculi muscle plays an important role in eyelid closure, affecting tear distribution and tear pumping [39, 40]. Mak et al. [21] described that in upper blepharoplasty usually only a small strip of preseptal orbicularis is excised and since the pretarsal portion is the part that plays a bigger role in blinking, this surgical excision has a limited impact on blink dynamics. Symptoms of dry eye lasting more than a week can be caused by a weakened orbicularis muscle and usually resolve with time. If these symptoms become persistent, the patient is considered to have dry eye syndrome [39, 40]. It has also been described that dry eye symptoms can be alleviated after upper blepharoplasty due to the improvement of eyelid function and corneal irritation after excess skin removal [22, 39].

Regarding dry eye tests, Schirmer testing measures tear film production, [42] tear film break-up time (TF-BUT) assesses tear film stability [22, 31, 42] and the ocular surface disease index (OSDI) is a questionnaire that assesses patient-reported dry eye symptoms [22].

In our systematic review, no studies reported a significant impact of upper blepharoplasty on the Schirmer 1 and 2 test. One study [21] found a significant increase and another one [22] found a significant decrease in the OSDI scores after surgery. Both studies performed skin and orbicularis removal. No significant differences in TF-BUT were found in our meta-analysis.

The authors suggest that since no difference was found in the tear film evaluation, the increase in dry eye symptoms reported by Mak et al. [21] could be attributed to the inflammation caused by the surgery.

Regarding aesthetic results, a few different approaches to upper blepharoplasty surgery have been described in this review and most patients were satisfied with the results. However, there is a need for more studies comparing different types of surgery and the different aesthetic results that can be achieved with each approach.

Common complications associated with upper blepharoplasty include edema, itching, and hematoma. One study found these symptoms to be higher in patients who underwent upper blepharoplasty with skin and orbicularis resection compared to patients who underwent skin resection only, on the seventh day postoperatively [12].

One study described a case of strabismus following upper blepharoplasty. Syniuta et al. [23] consider that a possible cause for this could be trauma to the superior oblique muscle, anesthetic toxicity, or inflammation from trochlear trauma. They also described that blind cauterization could lead to trochlear damage or lesions to the superior oblique muscles [23].

Some limitations to mention in this review are the lack of objectivity regarding the analysis of the aesthetic results, based on patient satisfaction, and the limited number of studies included about the functional impact of upper eyelid blepharoplasty.

The development of an objective and standardized method of evaluating upper eyelid blepharoplasty aesthetic results is in demand. This necessity becomes even more relevant when taking into consideration the numerous surgical techniques currently performed in plastic surgery and the need for an accurate method of comparing their results.

Recently, Arslan et al. [43] performed a study where they divided participants into groups according to the severity of dermatochalasis presented preoperatively. They considered the patients to have mild dermatochalasis when the upper eyelid skin barely connected to the eyelashes, moderate dermatochalasis when the upper eyelid skin overlapped the eyelashes, and severe dermatochalasis when the upper eyelid skin hung over the eye. This subdivision could be useful in future investigations as a standardized method of analyzing the impact of dermatochalasis and aging to the upper eyelid preoperatively, as well as upper blepharoplasty aesthetic results.

The authors consider that there is a necessity for a deeper understanding of the surgical impact on crucial functional parameters. The development of prospective randomized multicenter studies focusing on the true impact of upper eyelid blepharoplasty on CA, CCT, IOP, and dry eye symptoms, as measured by the Schirmer tests, TF-BUT and OSDI, would be extremely beneficial to the future of upper blepharoplasty surgery.

## Conclusions

Upper eyelid blepharoplasty was found to have no significant impact on visual acuity, intraocular pressure and dry eye symptoms in our meta-analysis. Regarding the aesthetic outcomes, most patients were satisfied with their results. In the studies included, the number of complications described was low.

To our understanding, there is a demand for an objective standardized method of evaluating and comparing aesthetic results between different surgical techniques and for the development of prospective randomized multicenter studies focusing on the true impact of upper eyelid blepharoplasty on CA, CCT, IOP, and dry eye symptoms.

## Declarations

### Human and Animals Rights

This article does not contain any studies with human participants or animals performed by any of the authors.

## References

[1] Fagien S (2002). Advanced rejuvenative upper blepharoplasty: enhancing aesthetics of the upper periorbita. Plast Reconstr Surg.

[2] Altin Ekin M, Karadeniz Ugurlu S (2020). Prospective analysis of visual function changes in patients with dermatochalasis after upper eyelid blepharoplasty. Eur J Ophthalmol.

[3] An SH, Jin SW, Kwon YH, Ryu WY, Jeong WJ, Ahn HB (2016). Effects of upper lid blepharoplasty on visual quality in patients with lash ptosis and dermatochalasis. Int J Ophthalmol.

[4] Yang P, Ko AC, Kikkawa DO, Korn BS (2017). Upper eyelid blepharoplasty: evaluation treatment, and complication minimization. Semin Plast Surg.

[5] Soares A, Faria-Correia F, Franqueira N, Ribeiro S (2018). Effect of superior blepharoplasty on tear film: objective evaluation with the Keratograph 5M - a pilot study. Arq Bras Oftalmol.

[6] Ilhan C, Aydemir GA, Aydemir E (2022). Changes in intraocular pressure and ocular biometry after blepharoplasty. Aesthetic Plast Surg.

[7] Jindal K, Sarcia M, Codner MA (2014). Functional considerations in aesthetic eyelid surgery. Plast Reconstr Surg.

[8] Sozer SO, Agullo FJ, Palladino H, Payne PE, Banerji S (2010). Pedicled fat flap to increase lateral fullness in upper blepharoplasty. Aesthet Surg J.

[9] Massry GG (2011). Nasal fat preservation in upper eyelid blepharoplasty. Ophthalmic Plast Reconstr Surg.

[10] Nalci H, Hosal MB, Gunduz OU (2020). Effects of upper eyelid blepharoplasty on contrast sensitivity in dermatochalasis patients. Turk Oftalmoloji Dergisi-Turkish J Ophthalmol.

[11] Osaki TH, Osaki MH, Ohkawara LE, Osaki T, Gameiro GR, Melo LAS (2020). Possible influence of upper blepharoplasty on intraocular pressure. Ophthalmic Plast Reconstr Surg.

[12] Damasceno RW, Cariello AJ, Cardoso EB, Viana GA, Osaki MH (2011). Upper blepharoplasty with or without resection of the orbicularis oculi muscle: a randomized double-blind left-right study. Ophthalmic Plast Reconstr Surg.

[13] Zinkernagel MS, Ebneter A, Ammann-Rauch D (2007). Effect of upper eyelid surgery on corneal topography. Arch Ophthalmol.

[14] Bhattacharjee K, Misra D, Singh M, Deori N (2020). Long-term changes in contrast-sensitivity, corneal topography and higher-order aberrations after upper eyelid blepharoplasty: a prospective interventional study. Indian J Ophthalmol.

[15] Ramella V, Stocco C, Facchin F, Troisi L, Papa G, Arnez ZM (2018). How to make your life easier: blepharoplasty markings with microsurgical clamps. Plast Reconstr Surg Glob Open.

[16] Thomas CB, Pérez-Guisado J (2013). A new approach: resection and suture of orbicularis oculi muscle to define the upper eyelid fold and correct asymmetries. Aesthetic Plast Surg.

[17] Aydemir E, Aksoy Aydemir G (2022). Changes in tear meniscus analysis after ptosis procedure and upper blepharoplasty. Aesthetic Plast Surg.

[18] Har-Shai Y, Hirshowitz B (2004). Extended upper blepharoplasty for lateral hooding of the upper eyelid using a scalpel-shaped excision: a 13-year experience. Plast Reconstr Surg.

[19] Zarem HA, Resnick JI, Carr RM, Wootton GD (1997). Browpexy: lateral orbicularis muscle fixation as an adjunct to upper blepharoplasty. Plast Reconstr Surg.

[20] Vola ME, Lisboa R, Diniz ER, Pereira NC, Kanecadan RT, Forseto ADS (2021). Influence of upper blepharoplasty on intraocular lens calculation. Arq Bras Oftalmol.

[21] Mak FHW, Ting M, Edmunds MR, Harker A, Edirisinghe M, Duggineni S, Murta F, Ezra DG (2020). Videographic analysis of blink dynamics following upper eyelid blepharoplasty and its association with dry eye. Plast Reconstr Surg Global Open.

[22] Hollander MHJ, Pott JWR, Delli K, Vissink A, Schepers RH, Jansma J (2022). Impact of upper blepharoplasty, with or without orbicularis oculi muscle removal, on tear film dynamics and dry eye symptoms: a randomized controlled trial. Acta Ophthalmol.

[23] Syniuta LA, Goldberg RA, Thacker NM, Rosenbaum AL (2003). Acquired strabismus following cosmetic blepharoplasty. Plast Reconstr Surg.

[24] Fan R, Chan TC, Prakash G, Jhanji V (2018). Applications of corneal topography and tomography: a review. Clin Exp Ophthalmol.

[25] Muzyka-Woźniak M, Oleszko A, Grzybowski A (2022). Measurements of anterior and posterior corneal curvatures with oct and scheimpflug biometers in patients with low total corneal astigmatism. J Clin Med.

[26] Mohammadi SF, Khorrami-Nejad M, Hamidirad M (2019). Posterior corneal astigmatism: a review article. Clin Optom (Auckl).

[27] Kanclerz P, Khoramnia R, Wang X (2021). Current Developments in Corneal Topography and Tomography. Diagnostics (Basel).

[28] Cavas-Martínez F, De La Cruz SE, Nieto Martínez J, Fernández Cañavate FJ, Fernández-Pacheco DG (2016). Corneal topography in keratoconus: state of the art. Eye and Vision.

[29] Zeppieri M, Gurnani B (2022). Applanation Tonometry, StatPearls.

[30] Sweeney DF, Millar TJ, Raju SR (2013). Tear film stability: a review. Exp Eye Res.

[31] Thulasi P, Djalilian AR (2017). Update in current diagnostics and therapeutics of dry eye disease. Ophthalmology.

[32] Simsek IB, Yilmaz B, Yildiz S, Artunay O (2015). Effect of upper eyelid blepharoplasty on vision and corneal tomographic changes measured by pentacam. Orbit.

[33] Sommer F, Untch E, Spoerl E, Herber R, Pillunat LE, Terai N (2022). Effect of upper eyelid blepharoplasty on corneal biomechanical, topographic and tomographic parameters 4 weeks after surgery. Int Ophthalmol.

[34] Floegel I, Horwath-Winter J, Muellner K, Haller-Schober EM (2003). A conservative blepharoplasty may be a means of alleviating dry eye symptoms. Acta Ophthalmol Scand.

[35] Dogan E, Akbas Kocaoglu F, Yalniz-Akkaya Z, Elbeyli A, Burcu A, Ornek F (2015). Scheimpflug imaging in dermatochalasis patients before and after upper eyelid blepharoplasty. Semin Ophthalmol.

[36] Akidan M, Coban DT, Erol MK, Balci U (2020). Evaluation of visual field and balance function alterations in patients who underwent dermatochalasis surgery. J Ophthalmol.

[37] Francis BA, Varma R, Chopra V, Lai MY, Shtir C, Azen SP (2008). Intraocular pressure, central corneal thickness, and prevalence of open-angle glaucoma: the Los Angeles Latino Eye Study. Am J Ophthalmol.

[38] Sng CC, Ang M, Barton K (2017). Central corneal thickness in glaucoma. Curr Opin Ophthalmol.

[39] Belovay GW, Goldberg I (2018). The thick and thin of the central corneal thickness in glaucoma. Eye (Lond).

[40] Lelli GJ, Lisman RD (2010). Blepharoplasty complications. Plast Reconstr Surg.

[41] Mack WP (2012). Blepharoplasty complications. Facial Plast Surg.

[42] Zhang SY, Yan Y, Fu Y (2020). Cosmetic blepharoplasty and dry eye disease: a review of the incidence, clinical manifestations, mechanisms and prevention. Int J Ophthalmol.

[43] Arslan N, Kocamış S, Sabur H, Acar M (2022). Evaluation of the effect of dermatochalasis and upper eyelid blepharoplasty surgery on corneal epithelial thickness alterations. Aesthetic Plast Surg.

